# Assessment of virtual education during the covid-19 pandemic from the perspective of faculty members and students: a cross-sectional descriptive study in northwest Iran

**DOI:** 10.1186/s12909-023-04378-y

**Published:** 2023-06-02

**Authors:** Mohammad Heydari, Yalda Mousazadeh, Roghayeh Salmani, Esmaeil Mehraeen

**Affiliations:** 1 Department of Health Information Technology, Khalkhal University of Medical Sciences, Khalkhal, Iran; 2 Department of Public Health, Khalkhal University of Medical Sciences, Khalkhal, Iran; 3 Department of Midwifery, Khalkhal University of Medical Sciences, Khalkhal, Iran

**Keywords:** Covid-19, Virtual education, Assessment, University of Medical Sciences, Iran

## Abstract

**Background:**

Since the coronavirus outbreak, many countries have replaced traditional education with virtual education in order to prevent the disease spread, and also avoid stopping education. The aim of the present study was to assess the virtual education status at Khalkhal University of Medical Sciences during the covid-19 pandemic from the perspective of students and faculty members.

**Methods:**

This is a descriptive-cross-sectional study that was conducted between December 2021and February 2022. The study population included faculty members and students who were selected by consensus. Data collection instruments included demographic information form and a virtual education assessment questionnaire. Data analysis was carried out using independent T-test, one sample T-test, Pearson Correlation, and ANOVA test in SPSS software.

**Results:**

A total of 231 students and 22 faculty members of Khalkhal University of Medical Sciences participated in the present study. The response rate was 66.57%. The mean and standard deviation of assessment scores of students (3.3 ± 0.72) were lower than those of faculty members (3.94 ± 0.64), which showed a statistically significant difference (p < 0.01). User access to the virtual education system (3.8 ± 0.85) and lesson presentation (4.28 ± 0.71) obtained the highest scores from the perspective of students and faculty members, respectively. There was a statistically significant association between employment status and the assessment score of faculty members (p = 0.01), and the field of study (p < 0.01), the year of university entrance (p = 0.01), and the assessment score of students.

**Conclusion:**

The results showed a higher than mean assessment score in both groups of faculty members and students. There was a difference between faculty members and students in terms of virtual education scores in the parts that require the creation of better processes and more complete capabilities in the systems, which seems that more detailed planning and reforms will improve the process of virtual education.

## Background

Today, the Internet has created a suitable environment for virtual education systems [1]. Virtual education refers to instruction in a learning environment where teacher and student are separated by time or space, or both, and the teacher provides course content through course management applications, multimedia resources, the Internet, videoconferencing, etc. [2–4]. Challenges of the traditional system, the rapid development of the web network as an underlying and potential factor of online courses, benefits of electronic education and the budgetary limitations have provided a significant incentive for universities to grow and develop virtual education [5].

Since the outbreak of coronavirus, fundamental changes have been made in the global educational system. Covid-19 pandemic has caused the closure of schools and universities in many countries. To fight the coronavirus disease, some countries replaced traditional education with distance education [6]. All these changes were made with the aim of dealing with the crisis, preventing the spread of the disease, and avoid stopping education [7]. The implementation of virtual education during the Covid-19 crisis had differences compared to normal conditions, which include (a) suddenness: because it was used without prior preparation; (b) Being imposed: virtual education was considered a luxury in many countries, but it was used as a necessity under crisis situations; (c) Internationality: virtual education intervention was used as a non-pharmacological all over the world and formed a global reality. (d) Reputation: This has become a common interest in societies and dominates the public domain. (e) Universality: Before the covid-19 pandemic, virtual education was mostly used in universities; but today, it has reached schools and has become an educational tool in all educational levels and educational centers, ranging from preschool to Doctor of Philosophy (PhD) levels due to this disease [7].

So far, many studies have interpreted the benefits of virtual and electronic education. Direct learning and communication with learners through computers and the Internet, focusing on a comprehensive learning view, creating a learner-centered system instead of a teacher-centered one, flexible learning, new and appropriate learning methods, reproducibility, compensating and fixing errors and problems have been mentioned as capabilities and benefits of virtual education [8]. Based on studies, the shortage of necessary software and hardware infrastructures, limit in bandwidth, the costs of Internet, organizational and cultural obstacles were seen as main barriers of the development of virtual education in developed countries at the beginning [9]. In a study on evaluation of virtual education during the Covid-19 pandemic, students considered flexibility and suitable teaching-learning platform as benefits and insufficient skills of virtual teaching by some faculty members, lack of effective learning, poor planning, invalid and incomplete evaluation, and hardware problems of the software system as one of the most important disadvantages of this type of education [10].

Assessing virtual education from various aspects is one of the important issues that can help identify existing problems, because effective solutions can be presented by identifying problems, barriers and weaknesses [11, 12]. Considering the foregoing, and since no studies have been conducted with the aim of assessing virtual education in Khalkhal University of Medical Sciences on the one hand, and there are few studies in Iran are on the other hand, the aim of the present study was to assess the status of virtual education in Khalkhal University of Medical Sciences during the covid-19 pandemic from the perspective of students and faculty members.

## Methods

### Study design

This was a descriptive-cross-sectional study, which was conducted in order to assess the status of virtual educational provided to students of Khalkhal University of Medical Sciences during the period of the covid-19 pandemic from the perspective of students and faculty members from December 2021 to February 2022.

Sampling and Data collection.

The study population included all 320 students of Khalkhal University of Medical Sciences and 60 faculty members who were selected by consensus method. Exclusion criteria included unwillingness to participate in the research project and incomplete questionnaires. Inclusion criteria also included people who had trained or taught at least one course online. After obtaining the necessary permissions from the Khalkhal University of Medical Sciences, data was collected using the electronic questionnaires prepared and sent to the students and faculty members through email and available virtual networks. The participants were give at least fourteen days to complete questionnaires.

### Instruments

Two questionnaires were used in the present study. The first questionnaire consisted of two parts including demographic characteristic and connecting devices and attendance in the system and virtual class. Some questions of this questionnaire were different between students and faculty members. Age, gender, field of study, year of entrance to university, place of residence, device of Internet connection, and how to participate in online class and exams were all questions from students. The faculty members’ questionnaire included age, gender, department, level of education, work experiences, employment status, how to connect and hold virtual classes, and how to handle students’ problems and questions.

The title of second questionnaire was “assessing virtual education from different aspects from the point of users”. This questionnaire was used in the study by Rastgarpour and Gorjizadeh [13] under the title “Assessment of the efficiency of e-learning courses in Tarbiat Modares University from the users’ point of view”. In the study of Rastgarpour and Gorjizadeh [13], the validity of the questionnaire was approved by experts. Also, reliability was confirmed by Cronbach’s alpha coefficient that was equal to 0.96. This questionnaire included 8 main sections and 53 questions. These 8 sections included access (6 questions), support (5 questions), tests and questions (4 questions), exercises and assignments (6 questions), information sources (5 questions), electronic content (9 questions), user interface (10 questions), lesson presentation (9 questions). This questionnaire is scored based on a 5-point Likert scale (excellent = 5, good = 4, moderate = 3, weak = 2 and very weak = 1) [13]. The lowest score of each section is 1 and the highest score was 5 in this questionnaire. The total score of the questionnaire is obtained from the mean score of the dimensions of the questionnaire and is a number between 1 and 5 [13].

### Statistical analysis

The collected data were analyzed using descriptive and inferential statistics in SPSS ver. 25. First, in order to confirm the normality of the data distribution, Kolmogorov–Smirnov test was used. Then, frequency, percentage, mean and standard deviation were reported in the descriptive section. In the inferential section, the one sample T-test was used for each dimension and the resulting mean score was compared with the number 3. Also, Independent T-test, and ANOVA were used to determine the association between demographic variables and assessment scores. In addition, Pearson Correlation was checked between questionnaire items.

## Results

A total of 231 students and 22 faculty members of Khalkhal University of Medical Sciences participated in the present study (The questionnaire was distributed among all 320 students and 60 professors). The response rate was 66.57%. 72.71% (n = 68) of students were female and 29.4% (n = 68) of them were studying nursing. 77.1% (n = 177) of participants lived in the city. The largest number of student respondents (38.5%) stated that they are non-native. A total of 48.5% (n = 112) of the students entered university in 2020. The mean and standard deviation of the students’ age was 23.38 ± 6.66. The results also showed that 50% (n = 11) of faculty members were working in the clinical sciences group. 45.5%(n = 10) of faculty members were sessional instructors. 63.6% (n = 14) of participating faculty members had a master’s degree. The mean and standard deviation of faculty members’ age and years of work experience were (38.23 ± 6.9), and (8.9 ± 9.61) years, respectively. The demographic characteristics of the study participants are presented in Table 1.

**Table 1:** Demographic characteristics of virtual education system users (students and faculty members)

Users	Variable		Frequency	Percent
Students	Gender	Female	168	72.7
		Male	63	27.3
	Field of study	Nursing	68	29.4
		Public health	61	26.4
		Environmental health	53	22.9
		Midwifery	25	10.8
		Nutritional science	24	10.4
	Place of residence	City	178	77.1
		Village	25	10.8
		Not mentioned	28	12.1
	Housing status	Native	26	11.3
		Non-native	89	38.5
		Not mentioned	116	50.2
	Year of entry	2020	112	48.5
		2019	89	38.5
		2018	28	12.1
		2017	2	0.9
Faculty members	Gender	Female	11	50
		Male	11	50
	Department	Clinical Sciences	11	50
		Basic science	5	22.7
		Islamic-thought and general courses	6	27.3
	Employment Status	Contractual-permanent	6	27.3
		Temporary (4 years)	6	27.3
		Sessional	10	45.5
	University Degree	PhD	8	36.4
		Masters	14	63.6
	Work experience	1–5 years	11	50
		6–10 years	6	27.3
		11–15 years	1	4.5
		16–20 years	0	0
		Up 20 years	4	18.2

A total of 56.2% (n = 130) and 61.5% (n = 142) of the students used smart mobiles to connect to virtual education systems, classes and online exams, respectively. Moreover, 54.5% (n = 126) of them had also used the mobile internet to connect to the internet, 84.5% (n = 195) of whom stated that mobile phones and SIM cards are supported by the 4th generation of mobile networks. A total of 63.3% (n = 14) of faculty members used laptops to attend virtual education classes and electronic learning systems. Faculty members also connected to the Internet and participated in virtual education systems mostly through high-speed home Internet (27.3%, n = 6) and internal college Internet (22.7%, n = 5). Also, 95.5%(n = 21) of faculty members stated that their mobile phone or SIM card supports the 4th generation of mobile phone networks. 36.4% (n = 8) of faculty members used simultaneous online classes for teaching, and 40.9% (n = 9) of them referred to PowerPoint & Voice as the most common methods of presentation. A total of 50% (n = 11) of the faculty members responded to the problems and questions of the students during the virtual education course through the formation of social network groups. The specifications of connecting devices and the way to participants attended the online class are presented in Tables 2 and 3.

**Table 2:** Specifications of connecting devices and attendance in the system and virtual class by students

Variable		Frequency	Percent
Communication device to connect to virtual education systems and attend to class	Smart mobile	130	56.2
	Laptop	29	12.5
	Desktop computer	13	5.6
	Tablet	2	0.86
	Mobile and laptop (simultaneously)	43	18.6
	Mobile and computer (simultaneously)	9	3.9
	Mobile and tablet (simultaneously)	2	0.86
	Mobile, laptop, computer (simultaneously)	3	1.3
Internet connection	Mobile phone Internet	126	54.5
	High speed home internet (ADSL^*^)	34	14.7
	Both (simultaneously)	71	30
Mobile phone and SIM card support from the 4th generation of mobile phone networks	Both	195	84.4
	SIM card only	18	7.8
	Mobile only	14	6.1
	None	4	2.2
How to participate in online exams	Smart mobile	142	61.5
	Computer or laptop	24	10.4
	Item number 1 and 2	59	25.5
	Internet cafe or relative’s house	1	0.4
	A combination of all the above	5	2.2

*ADSL: Asymmetric Digital Subscriber Line

**Table 3:** Specifications of connecting devices and attendance in the virtual class by faculty members

Variable		Frequency	Percent
Communication device to connect to virtual education systems and attend to class	Smart mobile	1	4.5
	Laptop	14	63.6
	Desktop computer	2	9.1
	Mobile phone and laptop (simultaneously)	3	13.6
	Mobile, laptop, computer (simultaneously)	2	9.1
Internet connection	Mobile phone Internet	4	18.2
	High speed home internet (ADSL)	6	27.3
	Internal college Internet	5	22.7
	A combination of all the above	7	31.8
Mobile phone and SIM card support from the 4th generation of mobile phone networks	Yes (both)	21	95.5
	No (none)	1	4.5
The method of holding a virtual class	Simultaneous online classes	8	36.4
	Uploading content in the Navid^*^ system	5	22.7
	Face-to-face class (internship)	1	4.5
	A combination of simultaneous online class and Navid	3	13.6
	A combination of all the above	5	22.7
How to present content and uploads in the Navid system	PowerPoint & Voice	9	40.9
	PDF (Portable Document Format) & Word File	7	31.8
	Video & Image	1	4.5
	A combination of all the above	5	22.7
How to handle students’ problems and questions during the virtual course	Email and phone call	2	9.1
	Forming social network groups	11	50
	The formation of the Navid group and system	3	13.6
	Holding an in-person troubleshooting class	1	4.5
	A combination of virtual group formation and in-person class	1	4.5
	A combination of all the above	4	18.2

* The virtual education system of Iranian universities of medical sciences

The answers given to the factors of each dimension of the eight-item assessment questionnaire are presented in Table 4. “Security level of user’s access to personal page” (access dimension), with mean and standard deviation (4.03 ± 0.83), and (4.36 ± 0.72) obtained the highest score from the perspective of students and faculty members, respectively. “The need for a lesson summary for students’ study” (information sources) with mean and standard deviation (2.8 ± 1.17) had the lowest score from the students’ perspective. “Possibility of adding information sources by students to the lesson” (information sources) with mean and standard deviation (3.41 ± 1.05) had the lowest score from the perspective of faculty members.

**Table 4:** Eight assessment criteria of virtual education along with users’ answers to each question

Dimension	Factors	Students					Faculty members					Mean ± SD	
		Excellent	Good	Moderate	Weak	Very weak	Excellent	Good	Moderate	Weak	Very weak	Student	Faculty member
Access	The level of security of user access to the personal page	68	117	34	10	2	11	8	3	0	0	4.03 ± 0.83	4.36 ± 0.72
	The possibility of accessing the website anytime - anywhere	49	116	55	9	2	10	9	3	0	0	3.87 ± 0.81	4.32 ± 0.71
	Internet speed to download lessons and upload assignments	33	95	74	23	6	8	7	7	0	0	3.55 ± 0.94	4.05 ± 0.84
	Can be used through normal computers	36	115	60	13	7	8	11	2	0	1	3.69 ± 0.9	4.14 ± 0.94
	No need for special user settings	54	118	48	8	3	9	10	2	0	1	3.92 ± 0.83	4.18 ± 0.95
	The existence of a suitable website as media and with specific media applications	36	116	66	10	3	8	7	6	1	0	3.74 ± 0.81	4 ± 0.92
Support	Technical user support for solving technical problems (offline and online)	22	91	73	40	5	9	5	7	1	0	3.37 ± 0.95	4 ± 0.97
	Training users for correct use	30	89	79	28	5	9	8	5	0	0	3.48 ± 0.94	4.18 ± 0.79
	Answer to frequently asked questions	27	77	85	28	14	9	4	9	0	0	3.32 ± 1.03	4 ± 0.92
	Providing documentation to guide users offline and online	28	108	74	14	7	6	6	8	2	0	3.59 ± 0.88	3.73 ± 0.98
	Determining the minimum technical specifications of user systems and informing them in time	29	96	80	20	6	8	7	7	0	0	3.53 ± 0.91	4.05 ± 0.84
Tests and questions	Pre-lesson test to know the students and their scientific background in the subject area	19	71	78	49	14	7	6	7	2	0	3.14 ± 1.03	3.82 ± 1.03
	Self-test quality at the end of each main part of the lesson (in accordance with the lesson objectives)	25	65	94	38	9	7	7	4	4	0	3.26 ± 0.98	3.77 ± 0.98
	The existence of different self-test in the course so that each small part of the course is accompanied by more than one question	16	77	85	39	14	7	8	3	4	0	3.18 ± 0.99	3.82 ± 0.99
	Designing suitable feedbacks for tests so that the user knows his/her weak points by observing and studying these feedbacks	28	77	80	35	11	7	11	2	2	0	3.33 ± 0.68	4.05 ± 0.68
Exercises and assignments	Individual exercises for each part of the lesson and providing appropriate feedback when students delivered their answers	20	71	97	34	9	7	9	5	1	0	3.26 ± 0.94	4 ± 0.87
	Group exercises for each part of the lesson and providing appropriate feedback after when students delivered their answers	25	68	97	30	11	6	9	4	3	0	3.29 ± 0.98	3.82 ± 1.006
	Definition of individual research and practical work	21	87	82	29	12	6	7	6	3	0	3.33 ± 0.98	3.73 ± 1.03
	Definition of group research and practical work	19	79	84	34	15	6	9	4	3	0	3.23 ± 1.03	3.82 ± 1.006
	Creating an environment for problem-solving and creativity	15	60	82	54	20	7	5	3	5	2	1.05 ± 2.98	3.45 ± 1.4
Information Resources	Electronic information resources needed for students to study and refer to in addition to the main course text	23	75	77	42	14	6	9	6	1	0	3.22 ± 1.05	3.91 ± 0.86
	A lesson summary for students to study	16	56	60	63	36	4	12	5	1	0	2.8 ± 1.17	3.86 ± 0.74
	List of websites relevant to the lesson and specify their connection with different parts of the lesson	25	66	86	45	20	4	10	3	5	0	3.09 ± 1.1	3.59 ± 1.05
	Determining the most important information sources for each part of the lesson	18	72	83	37	21	3	10	9	0	0	3.13 ± 1.06	3.73 ± 0.7
	Ability to add information resources to the course by students	18	61	84	44	24	3	9	4	6	0	3.02 ± 1.08	3.41 ± 1.05
Electronic content	The use of appropriate tests and exercises to present the lesson depending on the part of the lesson and the nature of the concepts	21	68	98	34	10	6	9	6	1	0	3.24 ± 0.96	3.91 ± 0.86
	Appropriate use of project definition, case study and problem solving to present lessons instead of direct presentation	17	73	93	38	10	4	10	6	2	0	3.21 ± 0.95	3.73 ± 0.88
	Clarifying the purpose of each part of the lesson and presenting it at the beginning of each part	24	89	87	22	9	5	13	4	0	0	3.24 ± 0.93	4.05 ± 0.65
	Lesson summary at the end of each lesson section	15	48	79	64	25	4	10	7	1	0	2.84 ± 1.07	3.77 ± 0.81
	Appropriate use of animation and multimedia capabilities to optimize content transfer	21	50	90	52	18	4	9	7	2	0	3.02 ± 1.05	3.68 ± 0.89
	Logical proportion between sound volume, image, text and animation	20	64	94	43	10	5	10	6	1	0	3.18 ± 0.97	3.86 ± 0.83
	The existence of a flexible environment for the user in order to control the sound, text and image and avoid creating an environment where the faculty member reads the lesson and turning the user into a listener-viewer	24	62	87	39	19	6	10	4	2	0	3.14 ± 1.08	3.91 ± 0.91
	The interactive nature of the lesson so that at least one meaningful interaction is created between the system and the user within seven minutes	16	58	98	45	14	6	10	5	1	0	3.07 ± 0.98	3.95 ± 0.84
	The existence of bibliography, terminology and important definitions separately for the whole or each important part of the course	18	72	86	39	16	4	7	7	4	0	3.16 ± 1.02	3.5 ± 1.01
User interface	Being able to access the course structure and information sources and other content structures with minimal clicks	23	73	104	21	10	6	10	4	9	0	3.34 ± 0.93	3.91 ± 0.92
	Existence of a fixed and meaningful rule for using numbers and bolts	20	68	102	30	11	5	9	4	3	1	3.24 ± 0.95	3.64 ± 1.13
	Use of appropriate colors in the design of pages and courses	30	80	84	31	6	5	11	5	1	0	3.42 ± 0.95	3.91 ± 0.81
	Using small-help messages like the help tool	25	79	92	25	10	6	8	6	2	0	3.36 ± 0.96	3.82 ± 0.95
	In lessons, there is fast movement between the previous and next pages and the first and last pages as much as possible	31	82	86	26	6	8	9	3	2	0	3.46 ± 0.95	4.05 ± 0.95
	The same format and structure in all parts of the course	23	91	84	27	6	7	12	2	1	0	3.42 ± 0.91	4.14 ± 0.77
	The ability to change the appearance of pages of the environment by the user without destroying other criteria	29	78	83	32	9	6	10	4	2	0	3.37 ± 1	3.91 ± 0.92
	Use of appropriate animation and graphics in lesson design	17	62	83	49	20	5	10	5	2	0	3.03 ± 1.06	3.82 ± 0.9
	Conventional fonts are used and there is a association between different text components such as type, shape, size and color of the fonts	28	74	83	34	12	6	11	3	2	0	3.31 ± 1.03	3.95 ± 0.89
	Possibility to access the information course structure and other content structures with a minimum number of clicks	27	75	92	30	7	6	12	3	1	0	3.37 ± 0.95	4.05 ± 0.78
Lesson presentation	Clarity of the faculty members’ schedule for attending and using chat environments, online and face-to-face classes	25	63	91	35	17	8	9	5	0	0	3.19 ± 1.05	4.14 ± 0.77
	Existence of strategy to use the forum as a central tool	21	61	91	41	17	8	6	6	2	0	3.12 ± 1.04	3.91 ± 1.01
	Continuous monitoring of students’ activities and supporting them according to their learning level	22	60	92	43	14	7	9	6	0	0	3.14 ± 1.02	4.05 ± 0.78
	Appropriate timing according to the content and course materials	22	53	98	44	14	6	13	2	1	0	3.11 ± 1.01	4.09 ± 0.75
	Timing of main exams	23	90	68	32	18	9	11	2	0	0	3.29 ± 1.07	4.32 ± 0.64
	Timing of exercises, projects and group activities	25	87	82	25	12	8	12	2	0	0	3.38 ± 0.99	4.27 ± 0.63
	Specific support notifications for each individual and group for better learning using mail, message and announcement tools to establish a suitable association with each group of students.	38	76	78	28	11	10	8	4	0	0	3.44 ± 1.05	4.27 ± 0.76
	Clarity of scoring method and calculation of the total course grade	20	73	81	38	19	10	9	2	1	0	3.16 ± 1.06	4.27 ± 0.82
	Distribution of the total course grade among all activities	23	76	92	28	12	9	10	3	0	0	3.31 ± 0.98	4.27 ± 0.7

The mean and standard deviation of students’ and faculty members’ assessment scores were 3.3 ± 0.72 and 3.94 ± 0.64, respectively. Independent T-test showed a significant difference between the opinions of students and faculty members regarding virtual education (p < 0.01). Users’ access to the virtual education system (3.8 ± 0.85) and lesson presentation (4.28 ± 0.71) obtained the highest scores from the perspective of students and faculty members, respectively. The mean score in all eight-item virtual education assessment questionnaire in the faculty member group was significantly different from the number 3 (Table 5). There was a statistically significant difference between the mean dimensions of users’ access to the virtual education system (3.8 ± 0.85), support for users of the virtual education system (3.48 ± 0.94), tests and questions (3.22 ± 0.92) and lesson presentation (3.31 ± 1.03) with the number 3 in the student group (Table 5).

**Table 5:** One sample t-test of the scores of dimensions of virtual assessment questionnaire from the perspective of faculty members and students

Variable	User	Number	Mean	Standard deviation	Means of standard error	Test value = 3
						Significance level
Access	Student	231	3.8	0.85	0.56	**< 0.01** ^*^
	Faculty member	22	4.17	0.85	0.18	**< 0.01** ^*^
Support	Student	231	3.48	0.94	0.62	**< 0.01** ^*^
	Faculty member	21	3.99	0.90	0.19	**0.002** ^*^
Tests and questions	Student	231	3.22	0.92	0.66	**0.01** ^**^
	Faculty member	22	3.86	0.92	0.21	**0.001** ^*^
Exercises and assignments	Student	231	3.21	0.99	0.65	0.16
	Faculty member	22	3.76	1.06	0.22	**0.02** ^**^
Information Resources	Student	231	3.05	1.09	0.72	0.21
	Faculty member	22	3.7	0.89	0.19	**0.01** ^**^
Electronic content	Student	231	3.11	1.02	0.06	**0.22**
	Faculty member	22	3.78	0.90	0.19	**0.006** ^*^
User interface	Student	231	3.3	0.99	0.06	0.13
	Faculty member	22	3.97	0.85	0.18	**< 0.01** ^*^
Lesson presentation	Student	231	3.31	1.03	0.06	**0.004** ^*^
	Faculty member	22	4.28	0.71	0.15	**< 0.01** ^*^

* P-Value < 0.01, ** P-Value < 0.05

Table 6 shows the association between the assessment score and demographic characteristics. ANOVA test showed a statistically significant difference between the scores of different groups of faculty members, including permanent, contractual, temporary, and sessional (P = 0.01, and F (3,18) = 5.05). The Least Significant Difference (LSD) post hoc test was used to perform all pairwise comparisons between group means. The results of this test showed that the mean scores of the temporary faculty members (4.33 ± 0.45) were significantly higher than the sessional, contractual, and permanent faculty members (P < 0.01).

**Table 6:** The association between faculty members’ demographic characteristics and the assessment score of virtual education

Variable		Number	Mean ± SD	T/F	Significance level
Gender	Female	11	3.79 ± 0.7	-1.06^*^	0.3
	Male	11	4.08 ± 0.57		
University Degree	PhD	8	3.78 ± 0.7	0.87^*^	0.39
	Masters	14	4.03 ± 0.61		
Department	Clinical Sciences	5	3.88 ± 0.72	0.65^**^	0.53
	Basic science	11	3.82 ± 0.75		
	Islamic-thought and general courses	6	4.2 ± 0.28		
Employment Status	Contractual-permanent	6	3.48 ± 0.52	5.069^**^	**0.01** ^***∞^
	Temporary (4 years)	10	4.33 ± 0.45		
	Sessional	6	3.74 ± 0.7		
Work experience	1–5 years	11	3.86 ± 0.65	1.7**	0.2
	6–10 years	6	4.3 ± 0.51		
	11–20 years	4	4.02 ± 0.63		
	Above 20 years	1	3.8 ± 0		
Internet connection	Mobile phone Internet	4	4.48 ± 0.6	2.25^**^	0.1
	High speed home internet (ADSL)	5	3.64 ± 0.76		
	Internal college Internet	6	4.14 ± 0.3		
	A combination of all the above	7	3.66 ± 0.63		

* Independent T-test **ANOVA *** P < 0.05 ∞ LSD post hoc

Table 7 shows the association between demographic characteristics of students and the assessment score of virtual education. ANOVA test showed a statistically significant difference between the assessment scores of environmental health, nutritional sciences, nursing, midwifery and public health (P < 0.01, and F (4,226) = 5.05). LSD post hoc test showed that the mean assessment scores of midwifery students (3.92 ± 0.7) were significantly higher than students in other study fields (P < 0.01). There was also a statistically significant difference between the assessment scores of the students according to year of entry (P = 0.01, F (3,227) = 3.8). LSD post hoc test showed that the mean scores of 2018 incoming students (3.01 ± 0.68) and 2109 incoming ones (3.2 ± 0.58) were significantly lower than 2020 those incoming ones (P < 0.01), and (P = 0.01) respectively.

**Table 7:** Association between students’ demographic characteristics and virtual education assessment score

Variable		Number	Mean ± SD	T/F	Significance level
Gender	Female	168	3.28 ± 0.72	-0.52^*^	0.6
	Male	63	3.34 ± 0.73		
Place of residence	City	178	3.3 ± 0.75	0.66^*^	0.5
	Village	25	3.2 ± 0.67		
Field of study	Environmental health	53	3.18 ± 0.61	6.35^**^	**< 0.01** ^***∞^
	Nutritional science	24	3.32 ± 0.57		
	Midwifery	25	3.92 ± 0.7		
	Nursing	68	3.13 ± 0.75		
	Public health	61	3.32 ± 0.73		
Year of entry	2020	2	3.44 ± 0.76	3.8^**^	**0.01** ^****∞^
	2019	28	3.01 ± 0.68		
	2018	89	3.2 ± 0.58		
	2017	112	3.45 ± 0.81		
Internet connection	Mobile phone Internet	126	3.26 ± 0.78	0.87^**^	0.41
	High speed home internet (ADSL)	34	3.44 ± 0.61		
	Both	71	3.31 ± 0.68		

* Independent T-test **ANOVA *** P < 0.01 **** P < 0.05 ∞ LSD post hoc

Table 8 shows significant positive correlations within all dimensions of assessment (p < 0.0001) among students. Regarding faculty members, different results were obtained. The dimension of access had a positive and significant correlation only with dimensions of support (r = 0.741, p = < 0.0001), and tests and questions (r = 0.444, p = 0.03). Two dimensions support, and test and questions had a significant positive correlation with all assessment dimensions (p = < 0.05). The results also showed each of the dimensions including exercises and assignments, information resources, electronic content, user interface, and lesson presentation had a significant positive correlation with all dimensions of assessment (p = < 0.05) except for the access.

**Table 8:** correlation between questionnaire items*

Variable	User	Variable							
		Access	Support	Tests and questions	Exercises and assignments	Information Resources	Electronic content	User interface	Lesson presentation
Access	Student	1	0.764^∞^ **< 0.0001** ^**¥**^	0.588 **< 0.0001**	0.583 **< 0.0001**	0.502 **< 0.0001**	0.541 **< 0.0001**	0.612 **< 0.0001**	0.583 **< 0.0001**
	Faculty member	1	0.741 **< 0.0001**	0.444 **0.03**	0.255 0.25	0.238 0.28	0.341 0.12	0.312 0.15	0.250 0.26
Support	Student	0.764 **< 0.0001**	1	0.774 **< 0.0001**	0.691 **< 0.0001**	0.696 **< 0.0001**	0.735 **< 0.0001**	0.716 **< 0.0001**	0.757 **< 0.0001**
	Faculty member	0.741 **< 0.0001**	1	0.777 **< 0.0001**	0.556 **0.007**	0.531 **0.01**	0.534 **0.01**	0.488 **0.02**	0.489 **0.02**
Tests and questions	Student	0.588 **< 0.0001**	0.774 **< 0.0001**	1	0.763 **< 0.0001**	0.739 **< 0.0001**	0.796 **< 0.0001**	0.691 **< 0.0001**	0.770 **< 0.0001**
	Faculty member	0.444 **0.03**	0.777 **< 0.0001**	1	0.759 **< 0.0001**	0.777 **< 0.0001**	0.716 **< 0.0001**	0.609 **0.03**	0.712 **< 0.0001**
Exercises and assignments	Student	0.583 **< 0.0001**	0.691 **< 0.0001**	0.763 **< 0.0001**	1	0.799 **< 0.0001**	0.804 **< 0.0001**	0.707 **< 0.0001**	0.751 **< 0.0001**
	Faculty member	0.255 0.25	0.556 **0.007**	0.759 **< 0.0001**	1	0.808 **< 0.0001**	0.790 **< 0.001**	0.719 **< 0.0001**	0.798 **< 0.0001**
Information Resources	Student	0.502 **< 0.0001**	0.696 **< 0.0001**	0.739 **< 0.0001**	0.799 **< 0.0001**	1	0.858 **< 0.0001**	0.711 **< 0.0001**	0.759 **< 0.0001**
	Faculty member	0.238 0.28	0.531 **0.01**	0.777 **< 0.0001**	0.808 **< 0.0001**	1	0.826 **< 0.0001**	0.728 **< 0.0001**	0.715 **< 0.0001**
Electronic content	Student	0.541	0.735	0.796	0.804	0.858	1	0.829	0.893
	Faculty member	0.341 0.12	0.534 **0.01**	0.716 **< 0.0001**	0.790 **< 0.0001**	0.826 **< 0.0001**	1	0.886 **< 0.0001**	0.929 **< 0.0001**
User interface	Student	0.612 **< 0.0001**	0.716 **< 0.0001**	0.691 **< 0.0001**	0.707 **< 0.0001**	0.711 **< 0.0001**	0.829 **< 0.0001**	1	0.833 **< 0.0001**
	Faculty member	0.312 0.15	0.488 **0.02**	0.609 **0.003**	0.719 **< 0.001**	0.728 **< 0.0001**	0.886 **< 0.0001**	1	0.826 **< 0.0001**
Lesson presentation	Student	0.583 **< 0.0001**	0.757 **< 0.0001**	0.770 **< 0.0001**	0.751 **< 0.0001**	0.759 **< 0.0001**	0.863 **< 0.0001**	0.833 **< 0.0001**	1
	Faculty member	0.250 0.26	0.489 **0.02**	0.712 **< 0.0001**	0.798 **< 0.0001**	0.715 **< 0.0001**	0.929 **< 0.0001**	0.826 **< 0.0001**	1

* Pearson Correlation,  ∞ The correlation coefficient (r), ¥ The significance level (P-value)

## Discussion

The present study assessed the status of virtual education at Khalkhal University of Medical Sciences during the Covid-19 pandemic from the perspective of students and faculty members. Assessing the status of virtual education provided from the perspective of student and faculty member users plays an important role in measuring the success rate of electronic education. The results of assessing the virtual education status can help to better identify the existing problems and lead to the improvement of programs, infrastructures and structures necessary for electronic education and learning. A more detailed explanation of the results and findings is mentioned below.

The results of the present study showed that the mean assessment scores of faculty members and students were higher than the mean value (number 3). However, students and faculty members had significantly different attitudes towards virtual education. The results of the present study also showed that all virtual education dimensions are favorable from the perspective of the faculty members. However, from the perspective of the students, the situation is favorable only in terms of access to the virtual education system, support for system users, tests and questions, and lesson presentation, but not other dimensions including exercises and assignments, information sources, electronic content, and the user interface. The results of the present study are consistent with the study by Seifi & Dibaie Saber. They also reported a statistically significant difference between the perspective of faculty members and students, and goal, content, access to educational and learning resources, faculty member-student interaction, evaluation of academic progress in electronic education were in favorable conditions from the perspective of faculty members but not students [14]. Esmaeili et al. showed that instructional content was undesirable from the point of perspective of students [15]. Jahanian & Etebar showed that the students participating in virtual E-learning centers were satisfied with the access to facilities of virtual training centers but, they did not have positive attitude towards virtual training [16]. Safdari et al. also found that quality of virtual education was above mean score from the point of perspective of faculty members and students [17].

It seems that the participants of this study are satisfied with the security and access to the virtual education network and the entire infrastructure provided for virtual education. Also, the main problems of students are related to the field of assignments, content, educational resources and presentation of courses. According to studies, there are five main groups that play an active role in any electronic education: authors, students, administrators, faculty members and teachers, as well as system experts [18, 19]. Also, technology and its infrastructure, content, instructor and learners, as well as learning methods can affect the effectiveness of courses and electronic and virtual education [20]. In a study, Mansouri Khosrowieh et al. referred to insufficient skills of faculty members in choosing and using media for teaching lessons, support and providing online advice, internet bandwidth problems and mismatch between existing curriculum and virtual education as challenges and harms of virtual education in the university during the Covid-19 pandemic [21]. According to the foregoing, the instructor plays a more prominent role in providing courses according to the virtual education environment. Empowering faculty members to use virtual education-related equipment, the instructor’s self-study in order to make virtual education targeted and effective, as well as revision of curricula and teaching and learning methods can be helpful in this regard. In their study, Kohpayehzadeh et al. stated that the use of appropriate methods for changes in educational environments and paying attention to the match between the distance learning environment and the needs of students and audiences and developing a strategy to improve the educational environment are of great importance and can improve the quality of education and learning in educational environments [22].

In this study, the question of “need for a lesson summary” got the lowest score from the students’ point of view. This indicates that the students expect the professor to provide a ready text of the summary of the taught material. The use of virtual education in developing societies such as Iran, which has different cultural and social values, causes users’ understanding of this type of learning to be different [23]. Many experts believe that virtual education has not been able to achieve all the basic goals of education and training, such as the development of creative thinking, commitment and responsibility, scientific risk-taking [24]. Sometimes students’ abilities affect their views. Unfamiliarity of nursing students with the Internet and computers was introduced as the most important barrier to holding online classes during the Covid-19 pandemic in a study in India [25].

The results of the study showed that the question “Possibility of adding information sources by students” got the lowest score from the faculty members’ point of view. This means that professors expected students to search and share more information. Shah Siah et al. point out in their study, the way of using and processing electronic information is considered a new and important factor for students in the electronic learning system and requires them to use a suitable method of searching and completing electronic information [26]. Keller et al.‘s study, by comparing the concerns of virtual professors of Argentine and Swedish universities, showed that compared to Swedish professors, Argentinian professors considered communication with students and active participation of students to be an important motivating factor. There are also problems such as lack of context creativity and the design of new ideas, lack of knowledge about technology, lack of motivational factors, and weakness of organizational culture were some of the obstacles in the educational experience of professors in virtual universities [27]. By comparing the studies, it seems that there is a need to establish clearer communication between professors and students and clarify their expectations.

The results of the present study showed that the highest response rate belonged to female gender, midwifery students and 2020 incoming students. However, there was a significant association only between field of study and year of entry with assessment score. The highest participation rate in the faculty member group belonged to the faculty members of the clinical sciences department and sessional faculty members. Also, most of the faculty members had a master’s degree. There was a statistically significant association between the employment status and the assessment score of faculty members. Rahban showed a significant decrease in satisfaction of quality of virtual education in all the studied dimensions with increasing age (faculty members and dental students) and years of work experience of the faculty members [28]. In a study on nursing students, Gaur et al. found a statistically significant difference in participants’ perspective regarding gender and year of entry, place of residence, father’s level of education, and family income [25]. Martha et al. identified significant differences in readiness for e-learning among students of Indonesia based on year of entry, field of study, cultural level, gender and region [29]. It seems that higher-semester students who had more experience in traditional education had a lower score for virtual education in the current study. Moreover, temprary faculty members compared to the permanent faculty members had a better evaluation of the virtual education status, which may be related to fewer working hours and less interaction with the virtual education system. On the other hand, since most of the midwifery students were employed, they preferred virtual education and obtained the highest assessment score.

The growth and development of electronic and digital technologies, along with the spread of the Covid-19 pandemic, has changed the way students interact with the educational environment. Technology-based learning (TBL) and distance education have replaced traditional education [7]. Smartphones are practical tools in electronic learning considering their portability, low price, quick and easy connection to the Internet [30]. The results of the present study show the wide use of smart mobile phones by students for electronic learning and attendance in virtual classes. These findings are consistent with the results of Al-Emran’s study in Oman [31]. Similarly, Heydari et al. and Mehraeen et al. confirmed that smartphones training applications were used for students and patients, and portable devices such as virtual reality headsets were popular devices to attend virtual education [32, 33].

The results also show that most faculty members chose simultaneous online classes for teaching, which is consistent with the study by McCrery et al. and Vezne’s study [34, 35]. Vezne found that extensive online courses had a positive impact on the personal growth and learning lives of teacher candidates [35]. It seems online courses are effective when student participation is provided. McCrery et al. argue that three following aspects of online courses influence how students engage with online discussions, and thus, learning opportunities: (a) the subject matter itself, (b) the representation and media through which that subject is engaged, and (c) online tasks [34]. Also, in order to upload educational content, faculty members often present their educational content through PowerPoint files along with audio files. In the problem-solving section, most of the faculty members emphasized that students use the capabilities of social networks such as WhatsApp to form question-and-answer groups and investigate students’ questions and problems. The use of groups in social networks such as WhatsApp, which was one of the methods of communicating with students by faculty members, is consistent with the results of Rahmadi’s study, in which the role of messenger and social network WhatsApp in education is mentioned [36].

Considering the foregoing, challenges of the traditional education system and Internet and the web network developments, the overall assessment has been effective and favorable, which is consistent with the results of the Mian’s study about student satisfaction with the telemedicine-based education [37]. The results also showed that faculty members were satisfied with all aspects of virtual education, and virtual education was satisfactory overall from the point of view of students, which are consistent with the results of Yazdanparast’s study at Bushehr University of Medical Sciences [38].

### Strengths and limitations

Considering that professors and students are the main beneficiaries of the educational system, getting their points of view regarding various aspects of virtual education can help to improve education. Another strength of this study was the use of a comprehensive questionnaire that was selected from several questionnaires that were used in Iranian universities. This questionnaire provided the possibility of a comprehensive investigation.The next strength is related to the application of the results of the present study. Since virtual education was implemented almost for the first time at the level of Iranian universities during covid-19 pandemic, the problems and challenges faced were the same. As a result, the identified problems and weaknesses can be used to solve problems in other universities as well. The results of the study were provided to the virtual education officials of the faculty and led to many reforms in the existing system.

Questionnaires were collected mostly through virtual networks and e-mail, and due to the lack of face-to-face communication, frequent reminders were usually needed. This study was only on a limited number of professors and students in a university of medical sciences. Therefore, the issue of generalizability may be questioned. As a result, different results may be obtained due to the difference in participants, scientific level of universities, and the level of facilities and equipment of different universities, as well as the cultural difference and the background of holding virtual education in those universities.

Considering that some time has passed since the pandemic, more studies are needed to identify the adaptability of users and the effectiveness of virtual education versus traditional education. Secondly, studies on a large number of students and professors are needed to generalize the results. On the other hand, it is suggested that the variables of culture, professors’ skills, and individual differences of students should be included in the studies as possible influencing variables.

## Conclusion

It seems that the movement towards electronic learning and virtual education that was formed before the Covid-19 pandemic and gained strength with the spread of such pandemic is welcomed by users. Despite its challenges, virtual education has also created special advantages from the users’ point of view, including distant education, attending the class from any place, reducing costs, facilitating access to faculty members and special courses regardless of location, an interactive environment with features such as providing an effective question and answer environment, presenting assignments and tests. The present study shows the positive view of faculty members towards virtual education and its systems, the general view of students is also positive and there was a difference only in parts of virtual education that require the creation of better processes and more complete capabilities in the systems. The movement towards virtual education during the Covid-19 pandemic, which affected many institutions and universities, should not be forgotten when the coronavirus pandemic ends. As mentioned, in addition to all the challenges and problems facing virtual education, it also has advantages. In addition to the educational systems, determining the quality level of the education provided by using new technologies requires more detailed studies.

## Data Availability

The datasets generated and/or analyzed during the current study are available from the corresponding author on reasonable request.

## References

[1] Moshtaghi S, Ogbehi A, Aghakasiri Z, Ahangari AH (2012). Evaluation of the virtual courses from students and Faculty Members of Khajeh Nasir Toosi University Viewpoints based on SCORM Standard. Educ Devel Jundishapur.

[2] Westbrook V (2006). The virtual learning future. Teach High Educ.

[3] Spicer JO, Nguyen TT, Arnold MW, Anderson T, Khalife R (2021). A Faculty Development Workshop for Planning and Implementing Interactive virtual case-based teaching. MedEdPORTAL.

[4] Moore RL, Fodrey BP. Distance education and technology infrastructure: strategies and opportunities. Leading and managing e-learning. Springer. 2018: 87–100.

[5] Yeung D (2001). Toward an effective quality assurance model of web-based learning. OJDLA.

[6] Jadhav VR, Bagul TD, Aswale SR. COVID-19 era: students? role to look at problems in education system during lockdown issues in Maharashtra, India. Int J Inf Res Rev. 2020; 7(5): 328–31.

[7] Jadhav VR, Bagul TD, Aswale SR (2020). COVID-19 era: students’ role to look at problems in education system during lockdown issues in Maharashtra, India. Int J Inf Res Rev.

[8] Eleftheriou A, Rokou A, Argyriou C, Papanas N, Georgiadis GS. Web-based medical education during COVID-19 lockdown: a step back or a leap to the future? Int J Low Extrem Wounds. 2022;21(3):272–4. 10.1177/15347346211011848 PMC9297065 33890811

[9] Golafshani SAH, Kebriaei A, Hoseindoost G, Arani MM, Ansaritabar A. Investigating the effective factors on tendency toward virtual education among the students of payame noor university in Kashan and Aran-Va-Bidgol county in 2017. Int Arch Health Sci.. 2020;7(2) 64–7.

[10] Arzani A. Exploration of the Students’ Perception to Virtual Education in Covid-19 Epidemic: A Qualitative Study. J Nurs Educ. 2021;10(1):91–104.

[11] Crisol Moya E, Herrera Nieves LB, Montes Soldado RA. Virtual education for all:Systematic review. Educ Knowl Soc. 2020; 21: 1–13.

[12] Radović-Marković M. Advantages and disadvantages of e-learning in comparison to traditional forms of learning. Ann Univ Petroşani, Economics. 2010;10(2):289 – 98.

[13] Rastgarpour H, Gorjizadeh S. Evaluating the effectiveness of e-learning courses at Tarbiat Modares University from the perspective of users. Int J Inf Commun Technol Educ Sci. 2012; 2(3): 5–30.

[14] Seifi R, Dibaeisaber M. Assessing the current status of the e-learning system of Shahed University during the Corona era from the perspective of professors and students. Curric Teach Perspect. 2021; 2(1): 8–13.

[15] Esmaeeli H, Rahmani S, Kazemi A, Ali Ahmadi M. Evaluation of E-Learning of the virtual learning program from the student’s point of view. Public Manag Res. 2016;9(34):203 – 22.

[16] Jahanian R, Etebar S. Evaluating the status of virtual education in e-learning centers of Iran’s universities from the viewpoint of students. Inform Comm Tech Edu Sci. 2012;2(4):53–65.

[17] Safdari MR, Shekari S, Jafari E, Roshanravan M, Namdar Ahmadabad H. Evaluation of Virtual Educations System from the Viewpoints of Faculty Members and Students in NKUMS during the Pandemic Coronavirus 2019. Horiz Med Educ Dev. 2021;12(2):96 – 81.

[18] O’Donoghue J, Singh G, Dorward L. Virtual education in universities: a technological imperative. Br J Educ Technol. 2001;32(5):511 – 23.

[19] Owolabi JO. Virtualising the school during COVID-19 and beyond in Africa: infrastructure,pedagogy, resources, assessment, quality assurance, student support system, technology,culture and best practices. Adv Med Educ Pract. 2020;11:755-9. 10.2147/AMEP.S272205 PMC7568587 33117046

[20] Alqahtani N, Innab A, Bahari G. Virtual education during COVID-19: Exploring factors associated with e-learning satisfaction among Saudi nursing students. Nurs Educ. 2021;46(2):E18-E22. 10.1097/NNE.0000000000000954 33234836

[21] Mansoury Khosraviyeh Z, Araghieh A, Barzegar N, Mehdizadeh A, Jahed H. Challenges and harms of e-learning at university during the Corona epidemic. Technol Educ J.2022; 16(4): 805 – 18.

[22] Koohpayehzadeh J, Afsharpour S, Naghizadeh Moogari Z. Psychometric Adequacy of The Persian Version of the DELES questionnaire to evaluate the educational environment of environment of Iran University of Medical Sciences. Razi J Med Sci. 2017;24(159):69–78.

[23] Kian M. Challenges of virtual education: a report of what are not learned. IJVLMS.2014;5(3):11–21.

[24] Dreyfus H. About the Internet: A Philosophical Look at the Internet. Farsinezhad A,trans.Tehran: Saghi Publications; 2010. p. 20–85.

[25] Gaur R, Mudgal SK, Dharni IT, Sharma R, Suyal N. Barriers encountered during online classes among undergraduate nursing students during COVID-19 pandemic in India. Int J Res Med Sci. 2020;8(10):3687-93.

[26] Shahsiah N, Nazarpouri A, Hakak M, Vahdati H. Providing an Strategic Electronic Learning Model for the Students of the Virtual Education Center at Isfahan University of Medical Sciences. Yafte. 2020; 21(4): 58–73.

[27] Keller Ch, Lindh J, Hrastinski S, Casanovas I, Fernandez G. The Impact of National Culture on Elearning Implementation: A Comparative Study of an Argentinean and a Swedish University. Educ Media Int. 2009;46(1):67–80.

[28] Rahban F. [Doctoral dissertation]. Survey of dental professors and students’ satisfaction with the quality of virtual education (year 1399) in Kerman University of Medical SciencesFaculty of Dentistry, Kerman University of Medical Sciences. Kerman: Kerman University of Medical Sciences; 2020.

[29] Martha ASD, Junus K, Santoso HB, Suhartanto H. Assessing undergraduate students’e-learning competencies: A case study of higher education context in Indonesia. Educ Sci. 2021;11(4):189.

[30] Artal-Sevil JS, Bernal-Agustín JL, Navarro JD. m-Learning (Mobile Learning) in Higher Education. The impact of smartphone as interactive learning tool. InEDULEARN15 Proceedings 2015 (pp. 8212–8221). IATED.

[31] Al-Emran M, Elsherif HM, Shaalan K. Investigating attitudes towards the use of mobile learning in higher education. Comput Hum Behav. 2016;56:93–102.

[32] Heydari M, Monagesh E, Mehraeen E, Aghamohammadi V, Seyedalinaghi S, Kalantari S, Nahid M, Nasiri K. Identifying data elements and key features of a mobile-based self-care application for patients with COVID-19 in Iran. Health Informatics J. 2021;27(4):14604582211065703. 10.1177/14604582211065703 34936526

[33] Mehraeen E, Noori T, Nazeri Z, Heydari M, Mehranfar A, Moghaddam HR, Aghamohammadi V. Identifying features of a mobile-based application for self-care of people living with T2DM. Diabetes Res Clin Pract. 2021;171:108544. 10.1016/j.diabres.2020.108544 33227362

[34] McCrory R, Putnam R, Jansen A. Interaction in online courses for teacher education:Subject matter and pedagogy. J Technol Educ. 2008;16(2):155 – 80.

[35] Vezne R. Teacher candidates’ satisfaction with massive open online courses in Turkey. Kıbrıslı Eğitim Bilimleri Dergisi. 2020;15(3):479 – 91.

[36] Rahmadi F. WhatsApp Group for Teaching and Learning in Indonesian Higher Education:What’s Up? Ijim. 2020; 14(13): 150-9.

[37] Mian A, Khan S. Medical education during pandemics: a UK perspective. BMC medicine.2020;18(1):1–2. 10.1186/s12916-020-01577-y PMC7141929 32268900

[38] Yazdanparast, A., Lashgari kalat, H., Marvi, N. A Survey of Medical Students’Opinions on the Quality of Virtual Education Courses Held in Bushehr University of Medical Sciences during the COVID-19 Pandemic. Med Edu Bull. 2020; 1(2): 77–86.

